# Hyoscine-*N*-Butyl-Bromide-Induced Hypotension and Myocardial Ischemia

**DOI:** 10.1155/2013/414856

**Published:** 2013-01-28

**Authors:** Guan-Liang Chen, Wen-Hsiu Hsu

**Affiliations:** ^1^Division of Cardiology, Department of Internal Medicine, Taichung Armed Forces General Hospital, Taichung 441, Taiwan; ^2^Division of Gastroenterology, Department of Internal Medicine, Taichung Armed Forces General Hospital, Taichung 441, Taiwan

## Abstract

Hyoscine *N*-butyl bromide, also known as scopolamine, is a type of antimuscarinic agent. This drug is associated with numerous common side effects, including abdominal fullness, constipation, urinary retention, blurred vision, skin flushing, tachycardia, decreased sweating, and salivation. The most unfavorable side effect is hemodynamic instability. In the present case, hypotension and acute myocardial infarction developed after intravenous hyoscine injection as a premedication therapy for colonoscopy. It was difficult to differentiate the cause-effect relationship between myocardial infarction and hypotension. Because both conditions were present under drug effects, we considered 2 possible diagnoses. One was coronary spasm with cardiogenic shock, and the other was myocardial ischemic sequela due to shock status. The latter diagnosis was confirmed after a series of examinations.

## 1. Introduction

Hyoscine *N*-butyl bromide, also known as scopolamine, is a type of antimuscarinic agent; this class of drugs also includes atropine, ipratropium, diphenhydramine, and others. Attachment of the butyl-bromide moiety prevents the movement of this drug across the blood-brain barrier, thereby minimizing its potential neurologic side effects. It is often used as an antispasmodic treatment for the pain and discomfort induced by abdominal cramps or menstrual cramps. Herein, we report a case of hypotension and acute myocardial infarction after intravenous hyoscine injection as premedication for colonoscopy. In this case, it was difficult to discern the cause-effect relationship between myocardial infarction and hypotension.

## 2. Case Report

**Figure 1:**
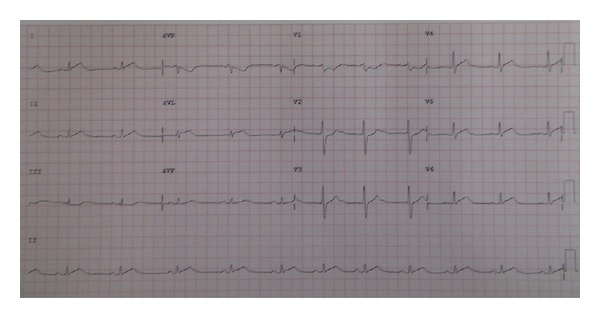
ST-segment elevation is revealed over the precordial V3−V6 lead.

A 53-year-old woman presented with abdominal cramping pain and bloody diarrhea for 3 days. She was robust prior to this event, with no known relevant medical history. She denied fever or chills. Physical examination revealed increased bowel sounds and tenderness in the lower abdomen. Laboratory tests demonstrated minimal leukocytosis (10,300 cells/*μ*L) without left shift and elevated C-reactive protein. Intravenous ciprofloxacin 400 mg q12 h was prescribed to treat the suspected infectious diarrhea. No specific finding was reported on enterogastroduodenoscopy. Due to suspicion of an infectious or inflammatory process of the lower gastrointestinal tract, colonoscopy was arranged after 3 days of colonic preparation. Hyoscine 20 mg was prescribed via a slow intravenous drip as a premedication for colonoscopy. Unfortunately, loss of consciousness and cyanosis of all 4 limbs developed 1-2 minutes later. The patient's blood pressure was 55/28 mmHg, and her heart rate was 58 beats per minute. After fluid resuscitation, epinephrine injection, and continuous dopamine infusion, the patient regained consciousness and her blood pressure recovered. However, electrocardiography revealed ST-segment elevation over the V3–V6 lead (Figure 1). Troponin I was elevated to 5.49 *μ*g/L.

**Figure 2:**
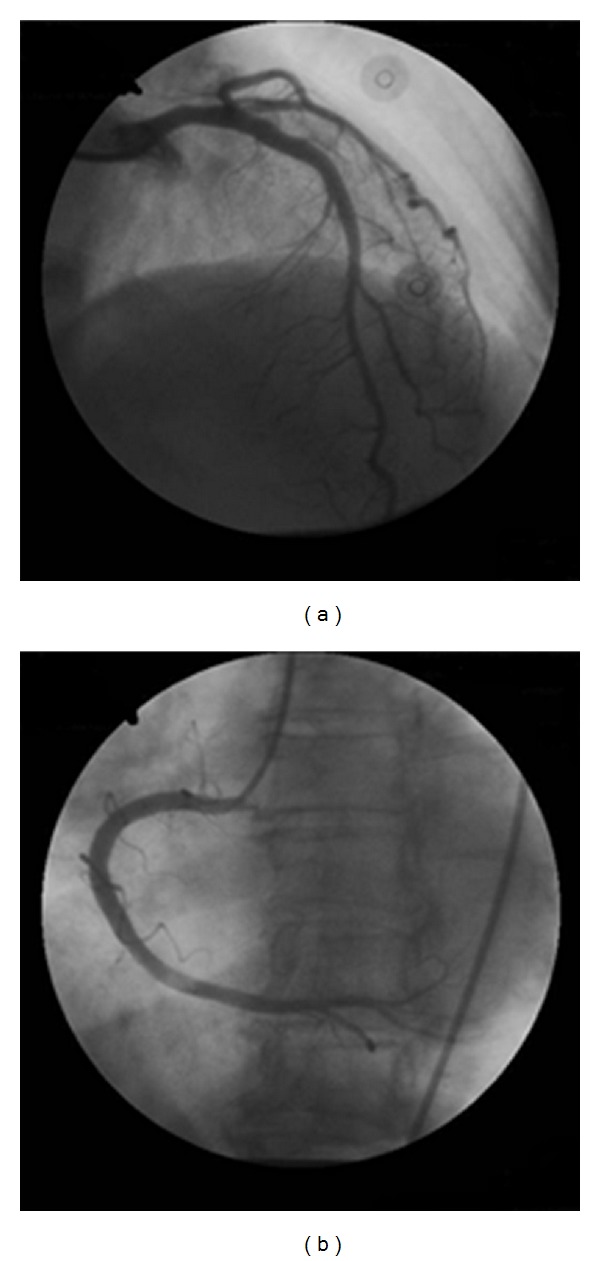
(a) Angiography of left coronary artery. (b) Angiography of right coronary artery. There is no obvious stenosis or obstruction in both images.

Emergent percutaneous coronary intervention was performed for ST elevation myocardial infarction. No obvious stenosis or obstruction was noted (Figure 2). Balloon angioplasty or stenting was not applied. Fluid hydration, heparinization, ticlopidine, and continuous dopamine were prescribed. On echocardiography, the ejection fraction and aortic wall motion remained normal. The dosage of dopamine and diuretics were tapered down, and the antiplatelet and anticoagulation agents were discontinued. Under conservative treatment with fluid hydration and antibiotics, the patient recovered gradually. The bloody diarrhea subsided, and no angina or heart failure signs were observed. The patient was discharged within 2 weeks.

## 3. Discussion

Hyoscine has been prescribed as a premedication therapy for colonoscopy to reduce discomfort and duration of the procedure in several studies [1, 2], although unfavorable results were reported in one randomized, controlled trial [3]. In addition, the benefits of this drug have not been reproduced in other studies [4, 5]. In clinical use, hyoscine is associated with numerous common side effects, including abdominal fullness, constipation, urinary retention, blurred vision, skin flushing, sedation, tachycardia, decreased sweating, and salivation. Its most unfavorable and rare side effect is hemodynamic instability. A case of hypotension and bradycardia was previously reported when hyoscine was combined with cyclopropane anesthesia [6].

Throughout the patient's course, there were 2 important clinical findings. The first was systemic hypotension, and the second was elevated troponin I. In addition, there were no remarkable findings on coronary angiography and echocardiography. According to above evidence, we considered 2 possible diagnoses. The first was coronary spasm with cardiogenic shock, and the second was myocardial ischemic sequela due to shock status. The mechanism of action of hyoscine involves antimuscarinic effects and smooth muscle relaxation. There have not been any reported side effects of coronary spasm or myocardial infarction. Hence, we tended to favor the other possible diagnosis. First, hypotension is clearly a reported side effect of hyoscine. Second, shock is a known cause of elevated troponin I. We reasoned that hyoscine first induced decreased vasal tone and shock. Then, elevated troponin I was demonstrated by the laboratory test because of myocardial ischemic sequela due to shock status, which is the cause of type 2 myocardial infarction according to the European Heart Journal classification [7]. Very few critical complications are reported with the common use of hyoscine, especially those such as hemodynamic instability or myocardial infarction. Whatever the definite cause, the initial management involves maintenance of blood pressure, antiplatelet agents, and emergent percutaneous coronary intervention. Cause and effect are needed to distinguish between diagnoses, as there are differences in the subsequent treatment strategies. Nitrate and calcium channel blockers are needed in patients with coronary spasm after hemodynamic stabilization. In contrast, hemodynamic maintenance and control of the underlying problem are the most important concerns for the treatment of shock patients. In conclusion, careful consideration of the use of intravenous hyoscine and preparation of resuscitation facilities should be considered due to the unpredictable risk associated with this medication in different individuals.
